# Surface deposited one-dimensional copper-doped TiO_2_ nanomaterials for prevention of health care acquired infections

**DOI:** 10.1371/journal.pone.0201490

**Published:** 2018-07-26

**Authors:** Tilen Koklic, Iztok Urbančič, Irena Zdovc, Majda Golob, Polona Umek, Zoran Arsov, Goran Dražić, Štefan Pintarič, Martin Dobeic, Janez Štrancar

**Affiliations:** 1 Department of Condensed Matter Physics, Jožef Stefan Institute, Ljubljana, Slovenia; 2 NAMASTE Center of Excellence, Ljubljana, Slovenia; 3 University of Oxford, John Radcliffe Hospital, The Weatherall Institute of Molecular Medicine, Human Immunology Unit, Headington, Oxford, United Kingdom; 4 Institute of Microbiology and Parasitology, Veterinary Faculty, University of Ljubljana, Ljubljana, Slovenia; 5 Department of Materials Chemistry, National Institute of Chemistry, Ljubljana, Slovenia; 6 Institute of Environmental and Animal Hygiene with Animal Behaviour, Veterinary Faculty, University of Ljubljana, Ljubljana, Slovenia; Louisiana State University Health Sciences Center, UNITED STATES

## Abstract

Bacterial infections acquired in healthcare facilities including hospitals, the so called healthcare acquired or nosocomial infections, are still of great concern worldwide and represent a significant economical burden. One of the major causes of morbidity is infection with *Methicillin Resistant Staphylococcus aureus* (MRSA), which has been reported to survive on surfaces for several months. Bactericidal activity of copper-TiO_2_ thin films, which release copper ions and are deposited on glass surfaces and heated to high temperatures, is well known even when illuminated with very weak UVA light of about 10 μW/cm^2^. Lately, there is an increased intrerest for one-dimensional TiO_2_ nanomaterials, due to their unique properties, low cost, and high thermal and photochemical stability. Here we show that copper doped TiO_2_ nanotubes produce about five times more ·OH radicals as compared to undoped TiO_2_ nanotubes and that effective surface disinfection, determined by a modified ISO 22196:2011 test, can be achieved even at low intensity UVA light of 30 μW/cm^2^. The nanotubes can be deposited on a preformed surface at room temperature, resulting in a stable deposition resistant to multiple washings. Up to 10^3^ microorganisms per cm^2^ can be inactivated in 24 hours, including resistant strains such as *Methicillin-resistant Staphylococcus aureus* (MRSA) and *Extended-spectrum beta-lactamase Escherichia coli* (*E*. *coli* ESBL). This disinfection method could provide a valuable alternative to the current surface disinfection methods.

## Introduction

Bacterial infections acquired in healthcare facilities including hospitals, the so called healthcare acquired or nosocomial infections (HAI), are still of great concern worldwide and represent a significant economical burden [1,2]. According to the World Health Organization antimicrobial resistance became an increasingly serious threat to global public health. Namely, many microbial species developed resistance to most newly discovered antibiotic, sometimes even before the drugs were introduced to the market [3,4]. Since the resistance occurs naturally over time, usually through genetic changes, it is inherently associated with the persistence of the bacterial colonization on the surfaces of the buildings, equipment, implants or tools. With its long time scale of many months [5,6], even up to a year [7], it can be thought as a possible long-term source of contamination and recurrent infections as it has been shown in case of medical implants [8]. To protect surfaces and avoid use of antibiotics at the same time, several approaches have been proposed. Silver or copper based antimicrobial surfaces were shown to be effective against different microorganisms, but their widespread usability remains questionable due to inherent copper toxicity to living cells [9], development of silver-resistant bacterial strains [10] and the unresolved environmental and health effects of released ions [11,12]. Even in the case of a more traditional disinfection strategies based on hydrogen peroxide H_2_O_2_ [13] or superoxide radical ·O_2_^−^ [14] cleaning, microbial resistance has been observed [15] with several enzymes responsible for that identified [16]. This is not surprising, since the ·O_2_^−^ radical and the H_2_O_2_ can be enzymatically degraded by superoxide dismutases (2·O_2_^−^ + 2H^+^ → H_2_O_2_ + O_2_) and catalases/peroxidases (2H_2_O_2_ → 2H_2_O + O_2_), respectively. On the other hand, no enzyme is known with ability to catalyze the ·OH [17], which can indiscriminately oxidize organic matter, including DNA [18], presenting a good base to prevent development of antimicrobial resistance. Interestingly, it has been shown that various types of efficient antibiotics, which attack different vital processes in bacteria, increase the production of ·OH hydroxyl radicals inside bacteria [19–21]. This suggests that antibacterial effect originates in oxidation caused by the ·OH radical. Taking into account the ability of various types of nanoparticles to produce ·OH radical and the possibility to deposit nanoparticles onto different surfaces, this would undoubtedly present a valuable means for surface antimicrobial protection.

Effectiveness of ·OH generation by TiO_2_ irradiated with metal halide lamp was demonstrated by Matsunaga et al. already in 1985, when *Escherichia coli* were completely sterilized [22]. Since then antibacterial effect of photo-excited TiO_2_ was shown against a wide range of bacteria, fungi, algae, protozoa, viruses, and even bacterial endospores, fungal spores, and protozoan cysts [23]. Based on the knowledge about photocatalytic mechanism and related photochemistry of TiO_2_ [24–27,27–31] (Figure A in S1 File) antibacterial action also depends on ·OH radicals, produced on the surface of photo-excited TiO_2_ with the energy of the light consistent with the TiO_2_’s bandgap (Figure A in S1 File, reaction ①). Although promising, photocatalytic disinfection of TiO_2_–based surfaces is not yet widely used. A recent review of commercially available antimicrobial nano-coatings in Europe showed that only one out of 23 antimicrobial coatings was based on the TiO_2_ [32]. With an average microbial density of several hundred per cm^2^ in common hospital surfaces [33] to several thousand per cm^2^ in drawers of frost-free home fridges (as conducted by Microban Europe (Cannock, Staffordshire) and European commission’s recommendation for “clean” food preparation and storage surfaces of less than 10 microbes per cm^2^, targeted disinfection efficiency is the inactivation of at least 10^3^ microbes/cm^2^. Indeed, this was achieved recently by Dunlop et al. [34] but at an activation with a rather high intensity UVA light of 30 W/m^2^. In another study, efficient suppression of growth of *Listeria monocytogenes*, which can cause dangerous foodborne illness with high mortality rate [35,36], could not be reached even at very high light intensities of up to 90 W/m [37]. We hypothesize that the main reason for such a failure is the same exceptionally high reactivity of the ·OH radical which is able to effectively degrade organic matter. Namely, due to ·OH radical decay by dimolecular reaction in which H_2_O_2_ is formed (Figure A in S1 File, reaction ②) [27]_,_ concentration of the ·OH radicals could significantly decrease with the distance from the TiO_2_ surface. A straightforward increase in photo-production of radicals due to higher intensity of light would thus not result in a stronger antimicrobial effect. For this one would needs to transform H_2_O_2_ back into ·OH radicals, for example by copper mediated Fenton type reaction [38,39] (Figure A in S1 File, reaction ③). An impact on photocatalytic properties of such a doping of TiO_2_ with transition metals such as copper has already been shown [40,41]. For example, several research groups made antibacterial coatings based on TiO_2_ containing copper and tested them at different light intensities [23,24,34,42–45]. With an antibacterial effect being not much different from the antibacterial effect achieved in the dark these studies indicate that much of the antibacterial effect is due to leached copper ions [42,43]. It turned out that higher amounts of copper than approximately 1% cannot be successfully incorporated into TiO_2_ structure, but are deposited as clusters on the TiO_2_ surface [46–48], from which copper can be released into solution. Although it contributes to toxicity of the nanoparticles in absence of irradiation [49] it leads to unacceptable development of copper resistant bacterial strains as well. Another challenge is application of TiO_2_ materials onto existing surfaces, since manufacturing process often requires high temperatures [34,42,44], thus makes it impossible to protect preformed, temperature sensitive materials. The efficient disinfection strategy should therefore rely exclusively on the incorporation of small amount of copper into TiO_2_ structure, which could be deposited onto a preformed surface, and activated by very low light intensities.

Recently, interest in one-dimensional TiO_2_ nanomaterials increased, due to their unique properties, low cost, and high thermal and photochemical stability [50]. Here we show that copper doped TiO_2_ nanotubes produce about five times more OH radiclas as compared to undoped TiO_2_ nanotubes and that effective surface disinfection can be achieved even at low intensity UVA light of 30 μW/cm^2^. The nanotubes can be deposited on a preformed surface at room temperature, resulting in a stable deposition resistant to multiple washings. Up to 10^3^ microorganisms per cm^2^ can be inactivated in 24 hours, including resistant strains such as *Methicillin-resistant Staphylococcus aureus* (MRSA) and *Extended-spectrum beta-lactamase Escherichia coli* (*E*. *coli* ESBL).

## Materials and methods

### Materials

The spin trap, 5-(Diethoxyphosphoryl)-5-methyl-1-pyrroline-N-oxide (DEPMPO) (Alexis, Lausen) was used as purchased without further purification and stored at -80°C. The spin-trap stock solutions were always freshly prepared. Ethanol (EtOH) and methanol (MeOH) from Merck AG (Darmstadt, Germany) were used in Lichrosolv® gradient grade quality. Titanium dioxide powder TiO_2_ (Degussa P25, Germany Degussa Corporation) and rutile TiO_2_ powder (Aldrich, Titanium(IV) oxide, rutile powder < 5μm) were dispersed in Erlenmeyer flask with KOH to obtain the stock solution of concentration 1 mg/ml. Media and culture materials were obtained from Gibco–Invitrogen Corporation (Carlsbad, California).

### Preparation of bacterial inoculum

Antimicrobial properties were tested on non-pathogenic bacterium *L*. *innocua*, which is closely related to pathogenic species *L*. *monocytogenes*, *E*. *coli*, *and S*. *aureus*. Suspension of each reference bacterial strain, was supplied by the Institute of Microbiology and Parasitology, Veterinary faculty, University of Ljubljana. Strain was maintained frozen at -70°C in sterile vials containing porous beads which serve as carriers to support microorganisms (Microbank, pro-lab Diagnostics) and kept at -70°C. The inoculum was prepared in liquid medium (Triptose soya broth, TSB, Biolife, Italia) and incubated aerobically for 24 h at 37°C. After incubation the culture contain approximately 10^9^ colony forming units (CFU) per milliliter. Working suspensions with appropriate concentrations were achieved by several 10-fold dilutions. The amount of the initial CFU/mL of the suspension placed in a petri dish was assessed by covering the suspension with approximately 15 mL nutrient agar. After overnight incubation at 37°C colonies were counted to estimate the CFU/mL in the original suspension.

### Synthesis and characterization of Cu-doped TiO_2_ nanotubes

Cu^2+^-doped TiO_2_ nanotubes (Cu-TiO_2_NTs) were prepared in several steps: (i) first sodium titanate nanotubes (Na^+^-TiNTs) were synthesized from anatase powder (325 mesh, ≥ 99.9%, Aldrich) and 10 M NaOH (aq) (Aldrich) at T = 135°C for 3 days under hydrothermal conditions. Exact synthesis procedure is described previously [51], (ii) in the next step Na^+^-TiNTs were rinsed with 0.1 M HCl(aq) yielding protonated titanate nanotubes (H^+^-TiNTs), (iii) then H^+^-TiNTs were dispersed in 0.5 mM solution of Cu^2+^(aq) (source of the Cu^2+^ was CuSO_4_·5H_2_O (Riedel de Haen)) and stirred at room temperature for 3 hours. By centrifugation the solid material was separated from the solution containing excess copper, and (iv) finally isolated material was dryed for 10 hours.

The powder X-ray diffraction (XRD) pattern was obtained on a Bruker AXS D4 Endeavor diffractometer using Cu Kα radiation (1.5406 Å; in the 2*θ* range from 10 to 65°). The elemental compositions of samples were investigated with a field emission scanning electron microscope (FE-SEM (Carl Zeiss, Super 35 LV) equipped with an EDX (energy dispersive X-ray spectrometer) element analysis system. Morphology of the particles in the sample was determined using transmission electron microscope (TEM, Jeol 2100). The specimen for the TEM investigation was prepared by dispersing the sample in MeOH with the help of an ultrasonic bath and depositing a droplet of the dispersion on a lacey carbon-coated copper grid.

### Photocatalytic activity of TiO_2_ nanotubes

The photocatalytic activity of synthesized TiO_2_ nanomaterials was determined using electron paramagnetic resonance spectroscopy (EPR) with spin trapping, which was optimized for measurement of primary radicals generated in the vicinity of the nanomaterial surface. This was achieved by measuring primary hidroxyl radicals in the presence of 30% ethanol with 5-(Diethoxyphosphoryl)-5-methyl-1-pyrroline-N-oxide spin trap (DEPMPO). EPR spin trapping was applied to measure the generation of reactive oxygen species (ROS) production. On the surface, small pool, proportionate to the size of the sample, was made with silicon paste and was filled with 2 μl of 0,5 M DEPMPO and 18 μl of 30% ethanol and irradiated with 290 nm diode for 5 min. The diode was 1–2 mm above the surface of the sample. The solution with short-lived radicals being trapped in the form of stable DEPMPO spin adducts was then drawn into the quartz capillary of 1 mm diameter, which was put in the 5 mm wide quartz tube and transferred into EPR spectrometer. All EPR measurements were performed on an X-band EPR spectrometer Bruker ELEXYS, Type W3002180. All measurements were recorded at room temperature using 1 Gauss (10^−4^ T) modulation amplitude, 100 kHz modulation frequency, 20 ms time constant, 15 x 20 seconds sweep time, 20 mW microwave power and 150 G sweep width with center field positioned at 3320 G.

### Deposition of Cu-TiO_2_NTs on surface and testing of the deposition stability

The deposition of Cu-TiO_2_NTs was made on polystyrene petri dishes (55.4 cm^2^). Petri dishses were washed before deposition. They were soaked in 20% NaOH solution, rinsed with distilled water, and finally with ethanol vapor. The suspension of the nanotubes with concentration of 1 mg/ml was processed with ultrasonic liquid processor (Sonicator 4000, Misonix) prior to the deposition on the slides. Sonication was performed using 419 Microtip^TM^ probe, 15 min process time, 10 s pulse-ON time, 10 s pulse-OFF time and maximum amplitude (resulting in 52 W of power). The petri dishes were treated with compressed air 3 times for 3 s. 150 μl of nanoparticle suspension was applied on each petri dish, immediately after compressed air treatment, and smeared evenly. The same number of dishes with nanoparticle deposition and control dishes were prepared for each experiment. On control dishes, only 150 μl of solution was applied. After the deposition, the dishes were left in the oven at 50°C for 2 hours. Then they were rinsed with distilled water and put back in the oven at 50°C for another 2 hours. The amount of deposited material was estimated from EPR signal decrease when soaking the surface with water, alkaline or acidic medium.

### Testing antimicrobial activity of Cu-TiO_2_NTs coated surfaces according to modified ISO 22196:2011

We tested antibacterial activity of Cu-TiO_2_NTs coated petri dishes (r = 8.4 cm, S = 55.4 cm^2^) using a slightly modified test according to standard ISO 22196 (Measurement of antibacterial activity on plastics and other non-porous surfaces), a 24-hour inoculation method [32], with different pathogens. We placed 1 mL of each bacterial suspension with different number of bacteria (from 100 to 10^5^ CFU/mL) in a petri dish for 24 hours at 4°C in a refrigerator. Petri dishes were covered with the lid to prevent drying of the suspension during the exposure. The surfaces were illuminated with a LED lamp (characteristics shown in the supplement, Figure F in S1 File) emmiting light at wavelength 360 nm and different light intensities as written in the main text of the manuscript. Next, bacterial suspension in each petri dish was covered with approximately 15 mL of agar at 37°C and incubated for 48 hours at 37°C, finally CFU were counted.

### Data reproducibility and statistics

**Fig 1:**
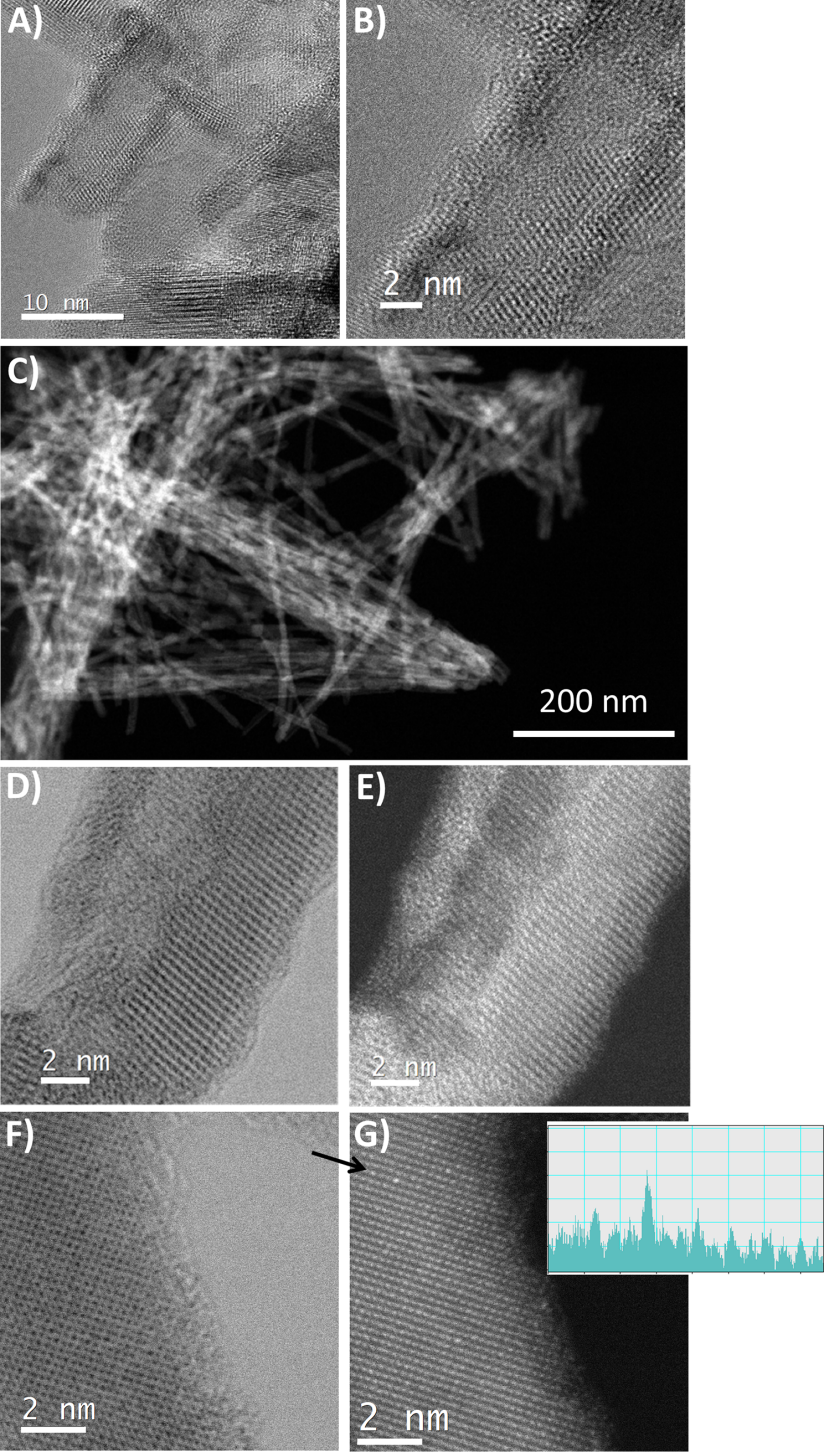
Transmission electron microscopy characterization of Cu-TiO_2_NTs. (A) and (B) high resolution transmission electron microscopy (HRTEM); (C) high-angle annular dark-field imaging scanning transmission electron microscopy (HAADF-STEM); (D) annular bright-field scanning transmission electron microscopy (ABF-STEM) and (E) HAADF-STEM of the same region; (F) ABF-STEM and; (G) HAADF-STEM of the same region of the CU doped TiO_2_ nanotubes with 1.2% of copper with the accompanying line intensity profile, acquired in direction indication by arrow.

**Fig 2:**
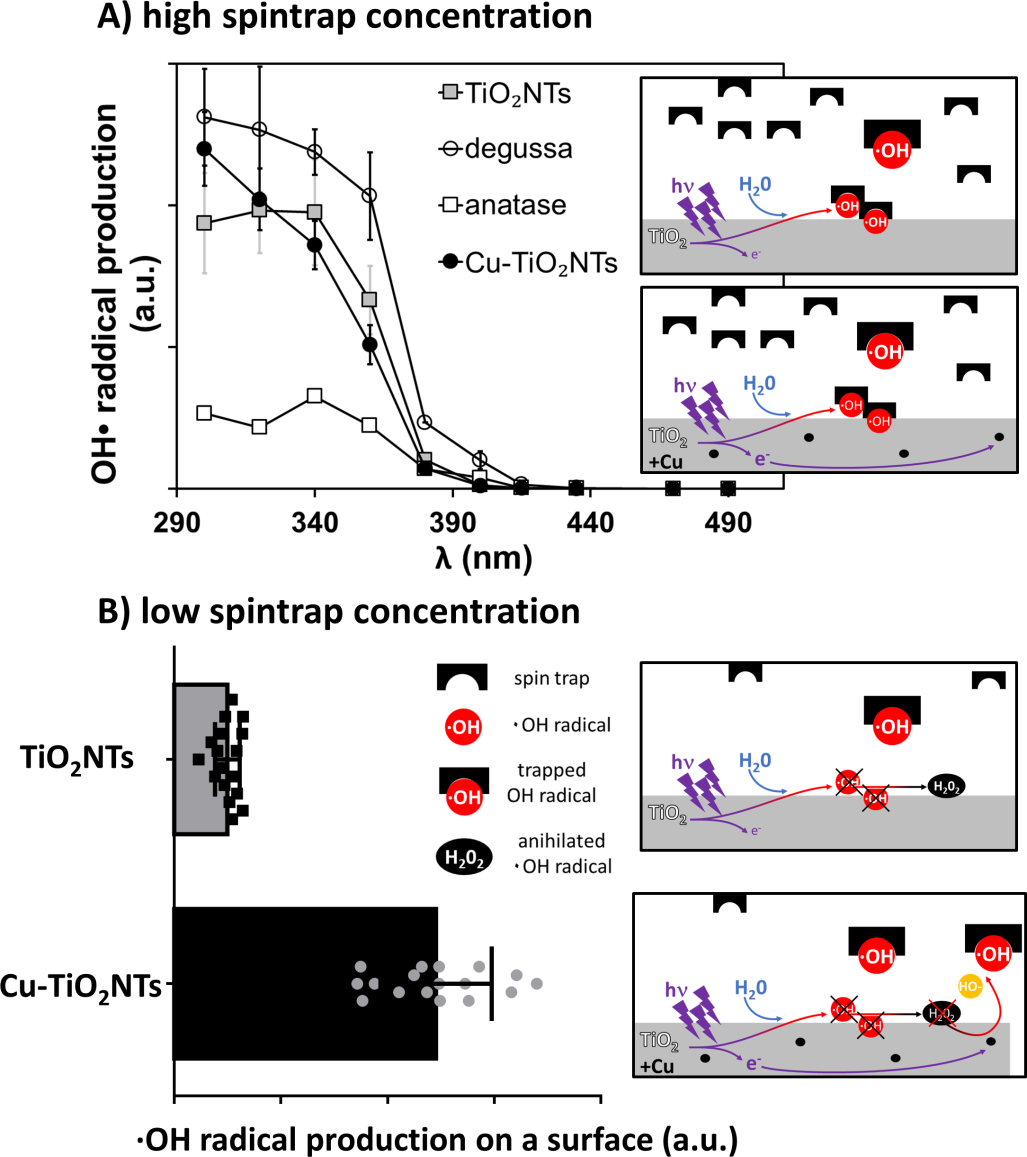
Photocatalytic activity of TiO_2_ nanotubes and TiO_2_ nanotube coated surface. A) Hydroxyl radical production at a TiO_2_ nanotube surface versus illumination wavelength measured at high concentration of the spin trap ethanol (17 M), data points are average values of multiple measurements, error bars represent standard errors; B) Hydroxyl radical production of a TiO_2_ nanotube coated surface measured at low concentration of spin trap DEPMPO (50mM) illuminated with a light emitting diode with peak wavelength of 320 nm. Height of the bars represent average values of 17 measurements (N = 17); error bars represent standard deviations. Each data point is also shown on the graph as closed squares for “TiO2NTs” series and as closed grey circles for “Cu-TiO2NTs” series.

HRTEM images in Fig 1 are representative images of the sample. Data points in Fig 2A are average values of multiple measurements; error bars represent standard errors. Each data point of the: 1) “TiO_2_NTs” series (Fig 2A, closed grey squares) was measured 2 times (N = 2), 2) “degussa” series (Fig 2A, open circles) was measured 2 to 3 times (N = 2–3), 3) “anatase” series (Fig 2A, open squares) was measured 1 time (N = 1), 4) “Cu-TiO_2_NTs” series (Fig 2A, closed circles) was measured 3 to 4 times (N = 3–4).

**Fig 3:**
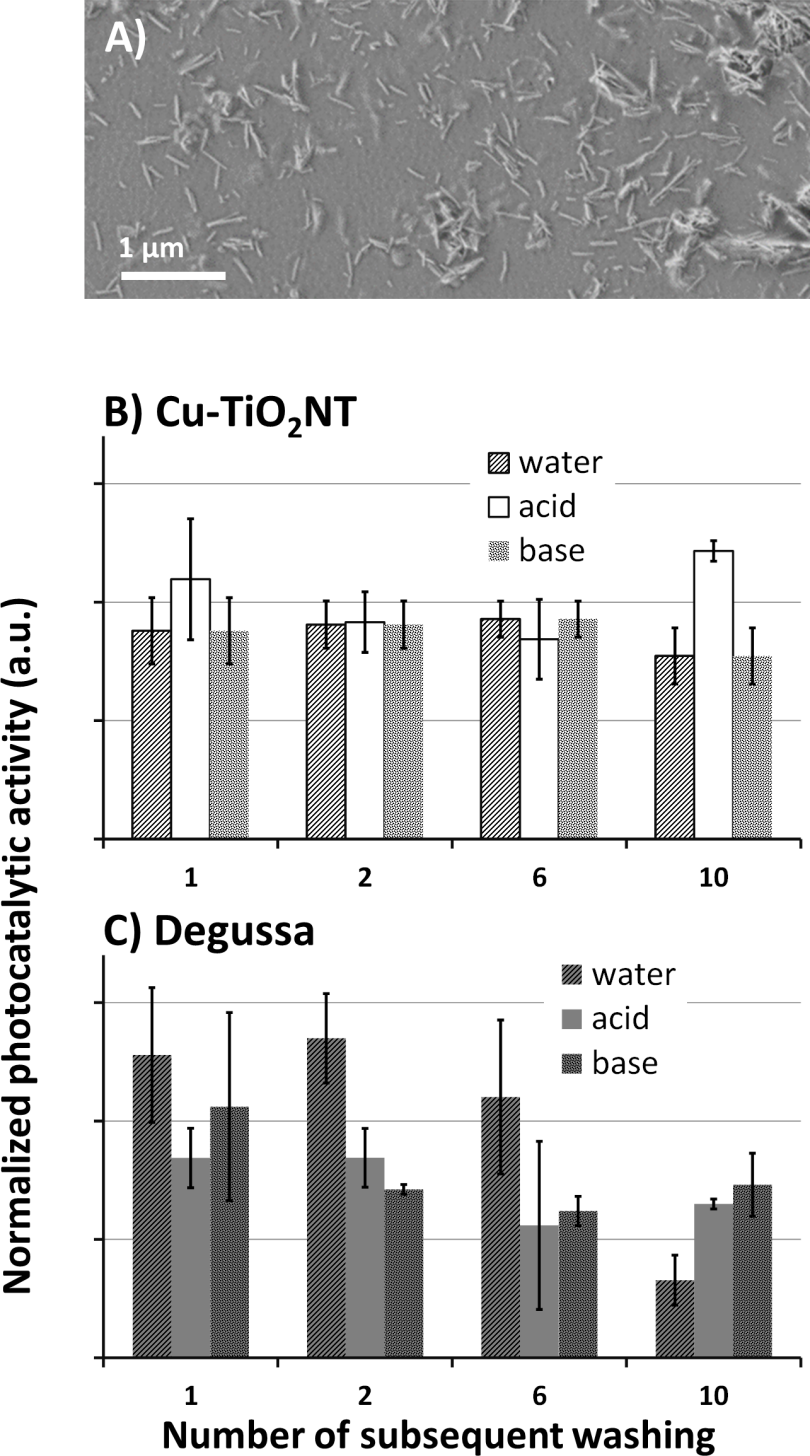
Deposition heterogeneity and stability of copper doped TiO_2_ nanotubes on a polymer surface. A) scanning electron microscopy image of TiO_2_ nanotubes deposited on a surface. B) relative activity of Cu^2+^-doped TiO_2_ nanotubes measured after several washing cycles at different pH and normalized to the activity measured before first washing. C) similar relative activity of the Degussa P25 deposition (water—bars with texture with diagonal lines, acid–bars with no texture, base–bars with dotted texture), without abrasion. Height of the bars represent average values of 6 measurements for each bar (N = 6); error bars represent standard deviations.

Height of the bars in Fig 2B represent average values of 17 measurements (N = 17); error bars represent standard deviations. Each data point is also shown on the graph as closed squares for “TiO_2_NTs” series and as closed grey circles for “Cu-TiO_2_NTs” series (Fig 2B). Height of bars in Fig 3B and 3C represent average values of 6 measurements for each bar (N = 6); error bars represent standard deviations.

**Fig 4:**
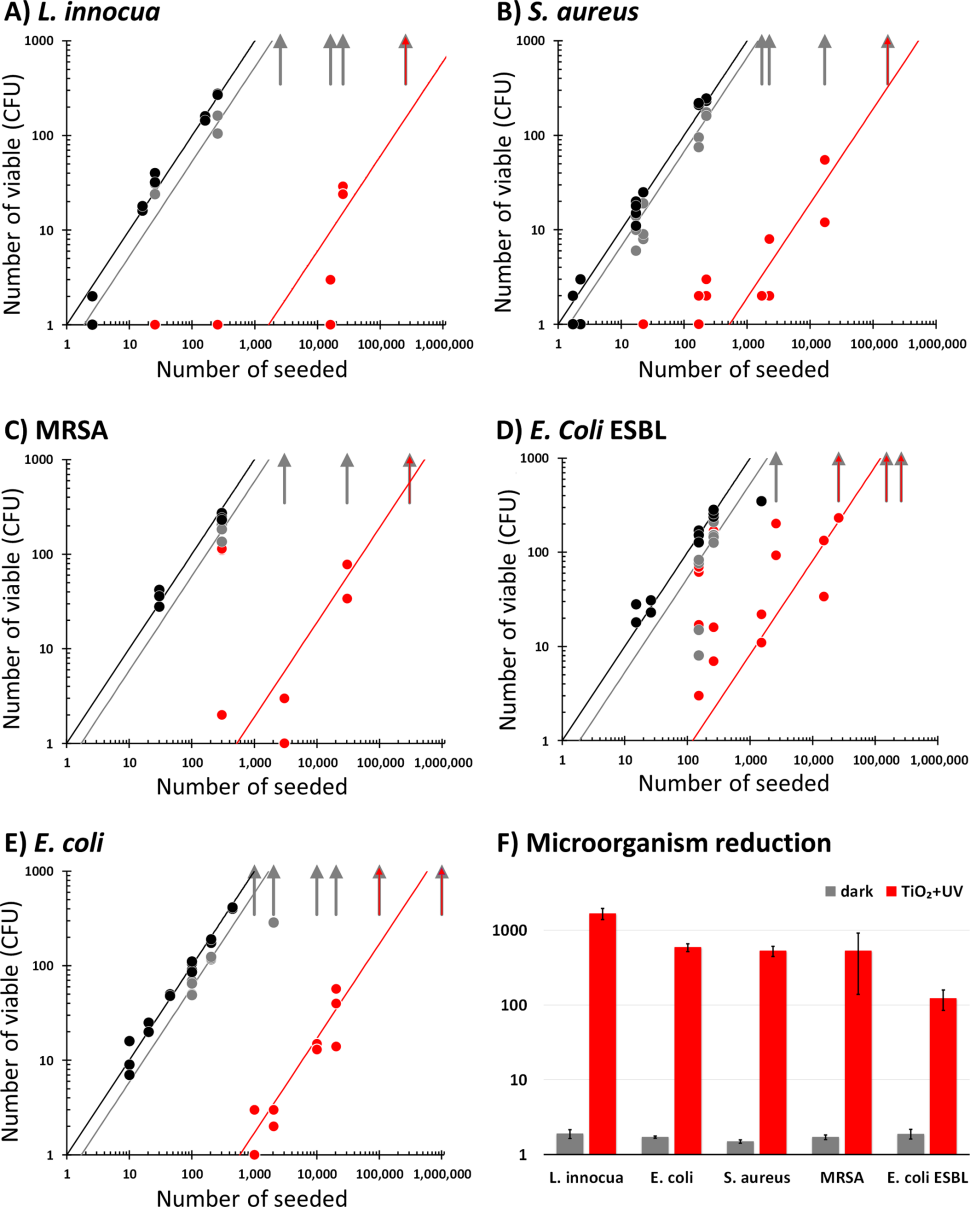
Antibacterial activity of Cu-TiO_2_NTs treated petri dishes against different microorganisms. (A) L. innocua; (B) S. aureus; (C) MRSA; (D) E. coli ESBL; and (E) E. coli. Number of colonies on control surfaces without the antibacterial coating kept in dark for 24 hours at 4°C in a refrigerator are shown as closed grey circles; Number of colonies on Cu-TiO_2_NTs coated petri dishes illuminated with 300 mW/m^2^ UVA light for 24 hours at 4°C in a refrigerator are shown as closed red circles; Number of colonies on control surfaces counted directly after inoculation are shown as closed black circles. Vertical arrows in each subframe of the figure represent the uncountable number of colonies (in our case set to 350) Straight lines through the data points represent best fit with only one parameter shown in frame F; (F) reduction of the number of microorganisms with regards to the number of inoculated microorganisms. Height of the bars represents value of regression coefficient *a* obtained by linear regression f = a*x through all the data points in each frame from A to E: 5 points (N = 5) for “*L*. *inncoua*” series, 9 points (N = 9) for “*S*. *aureus*” series, 6 points (N = 6) for “MRSA” series, 14 points (N = 14) for “*E*. *coli* ESBL” series, and 9 points (N = 9) for “*E*. *coli*” series. Error bars represent standard error of the regression coefficient.

Height of bars in Fig 4F represents value of regression coefficient *a* obtained by linear regression f = a*x through all the data points in each frame from A to E: 5 points (N = 5) for “*L*. *inncoua*” series, 9 points (N = 9) for “*S*. *aureus*” series, 6 points (N = 6) for “MRSA” series, 14 points (N = 14) for “*E*. *coli* ESBL” series, and 9 points (N = 9) for “*E*. *coli*” series; error bars represent standard error of the regression coefficient.

## Results and discussion

### Cu doped TiO_2_ nanotubes morphology and characteristics

The goal of this study was to produce TiO_2_ nanoparticles with high photocatalytic activity and small amount of copper ions incorporated into TiO_2_ lattice, avoiding leaching of the copper ions from the antibacterial surface. In addition, we wanted to develop TiO_2_ nanoparticles that would adhere better than spherical particles to achieve better stability on a preformed surface. Being aware that transition metal doping of titanate nanostructures results in a change of surface chemistry as well as in catalytic and photocatalytic properties [52], three different methods were considered [47]. Epitaxial growth of metal oxide phases on a surface was immediately excluded since it is more suitable for thin films [53] than for nanoparticle synthesis. On the other hand, ion exchange process using pristine alkali titanate nanostructures (ex situ doping) [52] and growth of sodium titanate nanotubes from already doped TiO_2_ (in situ doping) [54] produced mixed results: with an *in situ* doping it was shown that Cu^2+^ species may intercalate between titanate layers and preserve structure of the nanotubes, whereas in situ method does not produce well-formed nanotubes [47]. Moreover, ex situ doping at high levels of copper (app. 12%) produced nanotubes with Cu^2+^ species preferentially adsorbed on the surface of the nanotubes in the form of CuO nanoparticles, while doping at low levels of copper (app 0.02%) also produced well-formed nanotubes with more homogeneous distribution of Cu^2+^ ions. Although the distribution of Cu^2+^ could not be resolved by electron microscopy, low temperature electron paramagnetic resonance (EPR) method showed that 70% of EPR intensity could be attributed to intercalated Cu^2+^ ions, suggesting that around 30% of the Cu^2+^ ions were probably substitutionally doped into the titanate host structure [47].

In order to achieve high amount of incorporated copper ions into the TiO_2_ host structure, we prepared TiO_2_ and Cu^2+^-doped TiO_2_ nanotubes by calcination in air at 390°C (10 h) of pure hydrogen trititanate nanotubes (HTiNTs) and HTiNTs impregnated with Cu^2+^ [55,56]. XRD patterns of the TiO_2_ nanotubes (TiO_2_NTs) and Cu-doped^2+^ TiO_2_ NT (Cu-TiO_2_NTs) samples (Figure C in S1 File) correspond to anatase (ICDD No. 86–1157). We didn't observe any diffraction peaks belonging to CuO in the XRD pattern of Cu-TiO_2_NTs probably due to low copper content, 1.2 wt. %, as determined by EDXS analysis. Specific surface area of both samples, measured with BET analysis, is about 120 m^2^/g. During the transformation from hydrogen titanate to anatase, the nanotube morphology is retained (Figure D in S1 File), but some fragmentation can be observed. Nanotubes in both samples appear to be shorter than in the precursor HTiNTs [55] with the lengths being in the range between 100 and 200 nm and between 20 to 50 nm for the shortest fragments. The average nanotube diameter is about 9 nm, with the full range between 5 and 20 nm. Elemental analysis of the nanotubes confirm the uptake of Cu^2+^ into Cu-TiO_2_NTs that look slightly greenish (Figure C in S1 File, bottom) on the contrary to the TiO_2_NTs that are completely white (Figure C in S1 File, top).

### Cu doped TiO_2_ nanotubes high resolution electron microscopy

To resolve the exact incorporation of copper into the host structure we employed several advanced high resolution electron microscopy techniques. High resolution TEM (HRTEM) images show a crystalline structure of TiO_2_ nanotubes (Fig 1A and 1B) with no visible amorphous phase at the surface of the nanotubes. By applying a high-angle annular dark-field imaging scanning transmission electron microscopy (HAADF-STEM) we exclude the existence of larger CuO aggregates, since no obvious bright areas in “Z-contrast” imaging was detected (Fig 1C). Using annular bright-field scanning transmission electron microscopy (ABF-STEM), which enables to visualize atomic columns composed of light atoms, we observed thin (0.5 nm) amorphous layer at the surface of some nanotubes(Fig 1D), which is in agreement with XRD data discussed above (Figure C in S1 File), but no brighter contrast in HAADF-STEM (Fig 1E) indicating that no copper is present in this amorphous layer. Intensity distribution along the line indicated by black arrow and presented in the inset of Fig 1G brings us to conclusion that copper atoms indeed incorporate within the host matrix and do not aggregate on the surface of the TiO_2_ nanotubes.

We also performed electron energy loss spectroscopy (EELS) on the bulk and at the edge of a nanotube (Figure E in S1 File). Since the lower detection limit precludes copper detection in the nanotubes with only 1.2% of copper, it was applied to characterize Ti coordination. At the surface Ti is in lower coordination and possibly in Ti^3+^ state, whereas in the bulk we can see characteristic octahedrally coordinated Ti^4+^ EELS spectrum.

### Photocatalytic activity of TiO_2_ nanotubes

We tested the activity of the UV-excited nanotubes by measuring the concentration of photo-generated radicals using electron paramagnetic resonance (EPR) spectroscopy. Although this is a highly sensitive method for detecting paramagnetic species including radicals, some of them, like (and especially) ·OH radicals are so reactive that they never reach high enough concentrations to be detected by EPR. To overcome this, short-lived radicals must be trapped by nitrone compounds forming stable (long-lived) radical adducts, which can then be easily detected and their identity confirmed through their specific hyperfine splitting pattern. Although it has been shown that for some nanoparticles, such as graphene nanosheets, their photocatalytic activity depends on hydroxyl and superoxide radicals as well as holes [57], same is true for TiO_2_ nanoparticles, however, as shown by Kakuma et al. for anatase TiO_2_ the rate of ·OH generation is high, as compared with rutile TiO_2_, whereas the formation rate of ·O_2_^−^ for rutile is higher than that for anatase [58]. In this work we therefore used the spin trap 5-(dimethoxyphosphoryl)-5′-methyl-1-pyrroline N-oxide (DEPMPO), which is suitable for detecting superoxide radicals as well as hydroxyl radicals [59]. In agreement with the work by Kakuma et al. we identified that main radical produced on our anatase TiO_2_ nanotubes is the hydroxyl radical, whereas we could not detect the superoxide radical (Figure B in S1 File). By varying the spin trap concentration one can use this method to measure radical concentration on the nanoparticles’ surfaces, where radicals are generated, as well as in the bulk far from the nanoparticles’ surfaces, where radicals oxidize and kill microbes, i.e. where their effect makes a biological impact. In the first case much larger spin trap concentration is needed as compared to the second case. To achieve large enough concentration, we implemented the approach proposed by Adams et al. [60], by which ethanol is added to 30 vol% acting like an efficient and concentrated primary trap, which in addition transforms ·OH radical into a much more stable DEPMPO α-hydroxyethyl radical (CH(CH_3_)OH) spin adduct instead of DEPMPO-OH [61]. The analysis of the EPR spectra intensities reveals that the amount of ·OH radicals generated on the surface of the Cu^2+^-doped TiO_2_ nanotubes (Cu-TiO_2_NTs) is comparable to the amount generated by undoped TiO_2_ nanotubes (TiO_2_NTs) and almost as high as Degussa P25, commercially available spherical TiO_2_ nanoparticles (Fig 2A). The photocatalytic activity of the original material, from which the TiO_2_ nanotubes were synthesized, is, as expected, much lower (Fig 2A, open squares).

### Photocatalytic activity of a TiO_2_ nanotube coated surface

Although TiO_2_ and Cu-doped TiO_2_ nanoparticles generated similar amount of radicals (Fig 3A), the amount of radicals released from Cu-TiO_2_NTs far into the solution was approximately 5-times greater than that released from undoped TiO_2_NTs (Fig 3B). Since low spin trap concentration mimics rare targets like bacteria, which have not overcrowded surface yet, a copper-doped TiO_2_ nanomaterial provide much more efficient way for the photocatalytic surface disinfection. The presence of the copper atoms, well incorporated into the TiO_2_ matrix to prevent their release, transform H_2_O_2_, a product of the dimolecular reaction [27], back into more reactive ·OH radicals [38,39]. Although these results have been conducted with TiO_2_ nanotubes deposited on frequently used polystyrene surface via simple droplet deposition, washing and drying, the conclusions can be generalized to different surfaces. Nevertheless, despite the ability to adsorb nanoparticles, there is no other particular constraint on the protected surface.

### Deposition stability of Cu^2+^-doped TiO_2_ nanotubes on a polymer surface

Once proven that copper-doped TiO_2_ nanotubes are photochemically active, the deposition heterogeneity and stability were tested to identify the conditions of potential applications. Scanning electron microscopy revealed that Cu-TiO_2_NTs are fairly uniformly distributed on a polymer surface (Fig 3A). In addition, measurements of the photo-generated ·OH radical concentration after several consequent washings of the Cu-TiO_2_NTs deposited on a surface and illuminated with UVA light revealed a very stable and chemically-resistant deposition (Fig 3B). On the contrary, deposition of spherical nanoparticles Degussa P25 was found to be less stable (Fig 3C), which can be associated to different morphology of the nanoparticles with the tubes having larger adsorbing surface than the spheres.

### Antibacterial activity of Cu-TiO_2_NTs

Next, we tested antibacterial activity of Cu-TiO_2_NTs deposited on a polystyrene petri dish (r = 8 cm, S = 50cm^2^) using a slightly modified test ISO 22196, a 24-hour inoculation method [32], with different bacteria: *L*. *innocua*, *E*. *coli*, *E*. *coli* ESBL and *S*. *aureus* including MRSA. *L*. *innocua* has been chosen due to close relativity to *L*. *monocytogenes*, which has the highest mortality rate among all known foodborne pathogens and its ability to grew at very low temperatures, even at -1.5°C [62], common to food-storage rooms and fridges. Since *L*. *innnocua* is an example of Gram-positive bacteria, *E*. *coli* has been added to our selection as the very common Gram-negative bacteria. *S*. *aureus* has been included to the test as a very frequent strain that can cause many infectious with severe complications. In addition, two antibiotic-resistant bacterial strains *MRSA* and *E*. *coli* ESBL have been selected to check the efficiency against the bacteria that are currently among the biggest challenges in clinical medicine and maintenance of clean hospital infrastructure. Starting contamination concentration range between 10 and 10000 has been decided based on a typical number of bacteria per gram of food or cm^2^ of surface, which has then been renormalized to the surface of our petri dish. The petri dishes innoculated with the predefined number (as defined above) of the previously selected bacteria have been exposed and continuously illuminated with 300 mW/m^2^ at the same time for 24 hours at 4°C. Then they were covered with agar and incubated at 37°C for additional 3 days. The colony forming units (CFU) of microorganisms have been counted on petri dishes without the coating (Fig 4, grey circles) and on petri dishes with the Cu-TiO_2_NTs coating (Fig 4, red circles). To control the bacterial number in the inoculum, the CFU have been counted without the 24 hour UV exposure (Fig 4, black circles). The protection appeared to be very efficient for the majority of the bacterial strains, *L*. *Innocua* (Fig 4A), *S*. *aureus* (Fig 4B), MRSA (Fig 4C), and *E*. *coli* (Fig 4E) with the reduction in the number of inoculated microorganisms of about 10^3^ (Fig 4F). The lowest, but still good result was detected with *E*. *coli* ESBL (Fig 4D), where only about 100 microorganisms per selected petri dish surface could be completely inactivated in 24 hours. Additional controls as well as results after exposures to even smaller light intensities are shown in the supplement (Figure H in S1 File). Taking into account the common number of bacteria on the surfaces and that the doubling time of *Listeria* is more than a day at 4°C [63] (and much longer for other bacteria) such an antibacterial coating would definitively be capable of preventing cross contamination of foods or people getting in contact with different infrastructure parts. The data also show that neither illumination with UVA light of 200 mW/m^2^ nor coating of TiO_2_ nanotubes alone could effectivelly suppress the bacterial growth in the targetted range. Instead, one needs a combination of the tested nanomaterial-based coating and illumination of at least 50 mW/m^2^ of UVA light (Figure H in S1 File).

### Visualization and characterization of antimicrobial Cu-TiO_2_NTs coated surfaces with FMS

**Fig 5:**
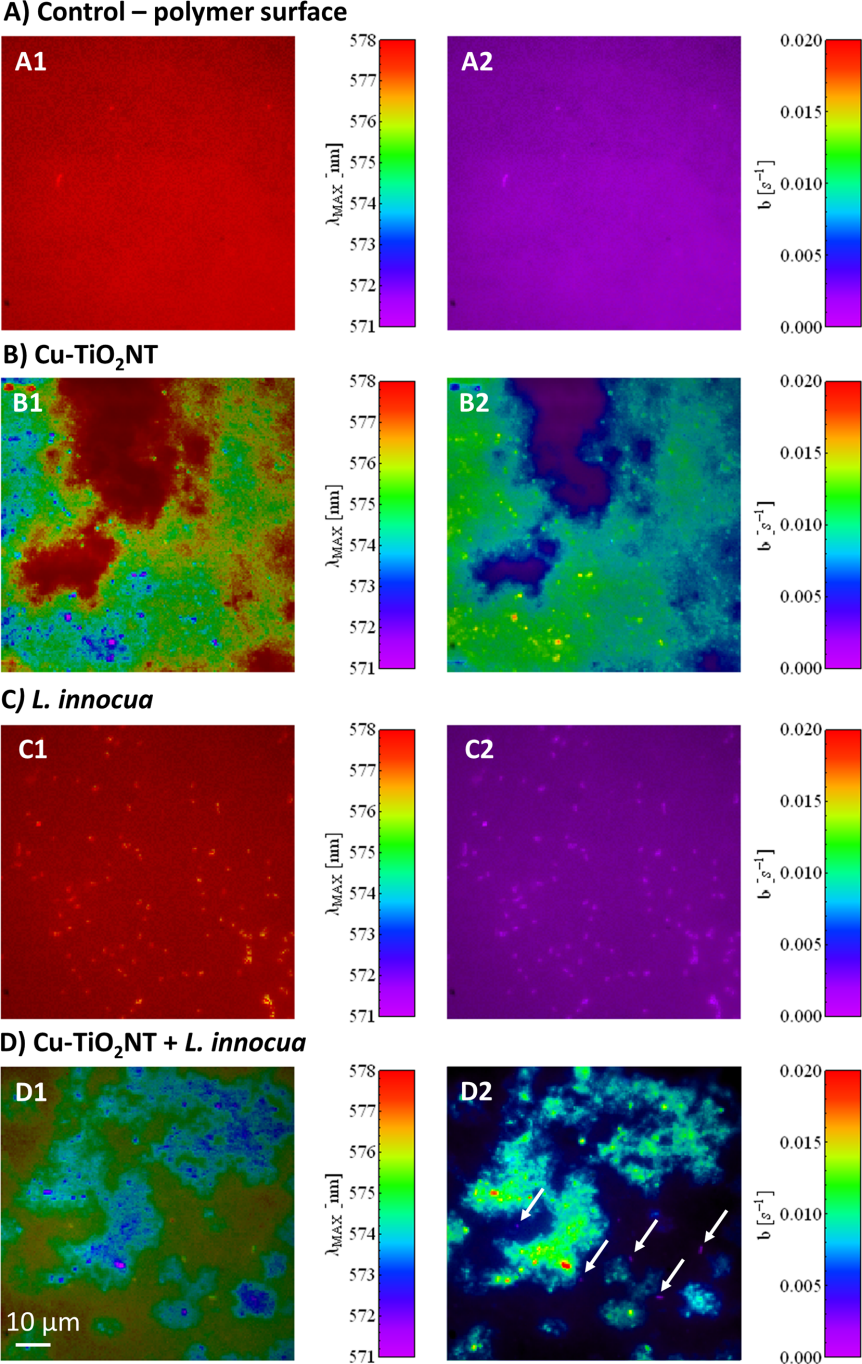
Characterization of Cu-TiO_2_NTs coated surfaces inoculated with *L*. *innocua* using bleaching-corrected fluorescence microspectroscopy. All samples were labeled with rhodamine B isothiocyanate (RITC). Column 1: wavelength of the fluorescence emission peak; and Column 2: bleaching rate images are shown for A) control—uncoated polymer surface, B) Cu-TiO_2_NTs coated surface, C) uncoated surface inoculated with *L*. *innocua*, and D) Cu-TiO_2_NTs coated surface inoculated with *L*. *innocua*. The spectrally contrasted images are color coded based on the local values of the associated fluorescence parameter (values for RITC spectral peak position (λ_MAX_) and bleaching rate (b), respectively). Lengths of the scale bars correspond to 10 μm on the sample. The white arrows mark individual bacteria that were identified by the spectral analysis.

To prove the relation between the disinfection efficiency and the contact/proximity between material and bacteria (because of high reactivity of ·OH radical), we tried to co-localize nanotubes and bacteria on the nanotube coated surfaces. To assess the later, we conducted microscopic visual inspection of inoculated surface (by *L*. *innocua* only, due to security reasons) that has been previously protected by deposition of tested nanomaterial. The whole sample (with nanotubes and bacteria on) has been stained by rhodamine B isothiocyanate (RITC), which strongly binds to proteins on the surface of bacteria and which at the same time enables identification of TiO_2_ nanotubes by their modulation of the RITC emission spectrum. Namely, fluorescence properties of rhodamine B are known to be strongly affected by TiO_2_ via self-photosensitized oxidative transformation [64], which accelerates its bleaching rate and induces a blue spectral shift [65]. In contrast, a red shift was expected when rhodamine molecules form oligomers due to re-absorption and re-emission of fluorescence light [66]—a phenomenon, which would be anticipated in the aggregates of dried probe on PET surface. The chosen dye therefore provides a unique opportunity to discriminate the native polymer surface, the TiO_2_ nanotubes and bacteria on the same spectrally-contrasted image (Fig 5), acquired by the spectrally-sensitive bleaching-corrected fluorescence microspectroscopy [67,68]. As anticipated, RITC fluorescence on the Cu-TiO_2_NTs coated surface showed remarkably lower fluorescence emission peak position wavelengths (Fig 5B1) and higher bleaching rates (Fig 5B2) compared to the RITC fluorescence on uncoated surfaces (Fig 5A). In the case when nanomaterial is spread non-uniformly, spectral shift can be used to localize nanomaterial more accurately than by using intensity image alone (Figure M in S1 File, frame B). Note, that bleaching rates (*b*) span very large range (Fig 5B2) and nicely anti-correlates with the wavelength of the emission maximum (Fig 5B1, λ_MAX_). Individual *L*. *innocua* cells were clearly identified on an uncoated surface by higher fluorescence intensity, very low bleaching rates as well as the blue-shifted spectral peak position (Fig 5C1). Thus, bacteria can be identified and counted also throughout the b- λ_MAX_ correlation graph (Figure M in S1 File, frame 5 C, red circle on the rightmost graph) as they are always located below the main population line that represent either uncoated polymer surface (at close-to-zero bleaching rate, black ellipsoid) or the TiO_2_ covered surface (at higher bleaching rates, pink elipsoid).

After innoculation with bacteria the TiO_2_-protected polymer surface FMS enables us to localize the bacteria much more reliably than with common fluorescence microscopy, where bacteria and nanoparticles’ aggregates look almost the same (Figure M in S1 File, frame 5 D, leftmost image). Both the wavelength-contrasted as well as the bleaching-rate-contrasted image clearly reveal that bacteria locate more probable on the part of surface without the coating (Fig 5D2, white arrows). Even though microscopy does not provide reliable statistics, a simple visual inspection supported the previously reported results that the coating significantly reduces the number of bacteria. Moreover, no clusters were detected with only few individual bacteria remaining on the areas with sparser coating.

### High-resolution visualization of antimicrobial Cu-TiO_2_NTs coated surfaces with SEM

**Fig 6:**
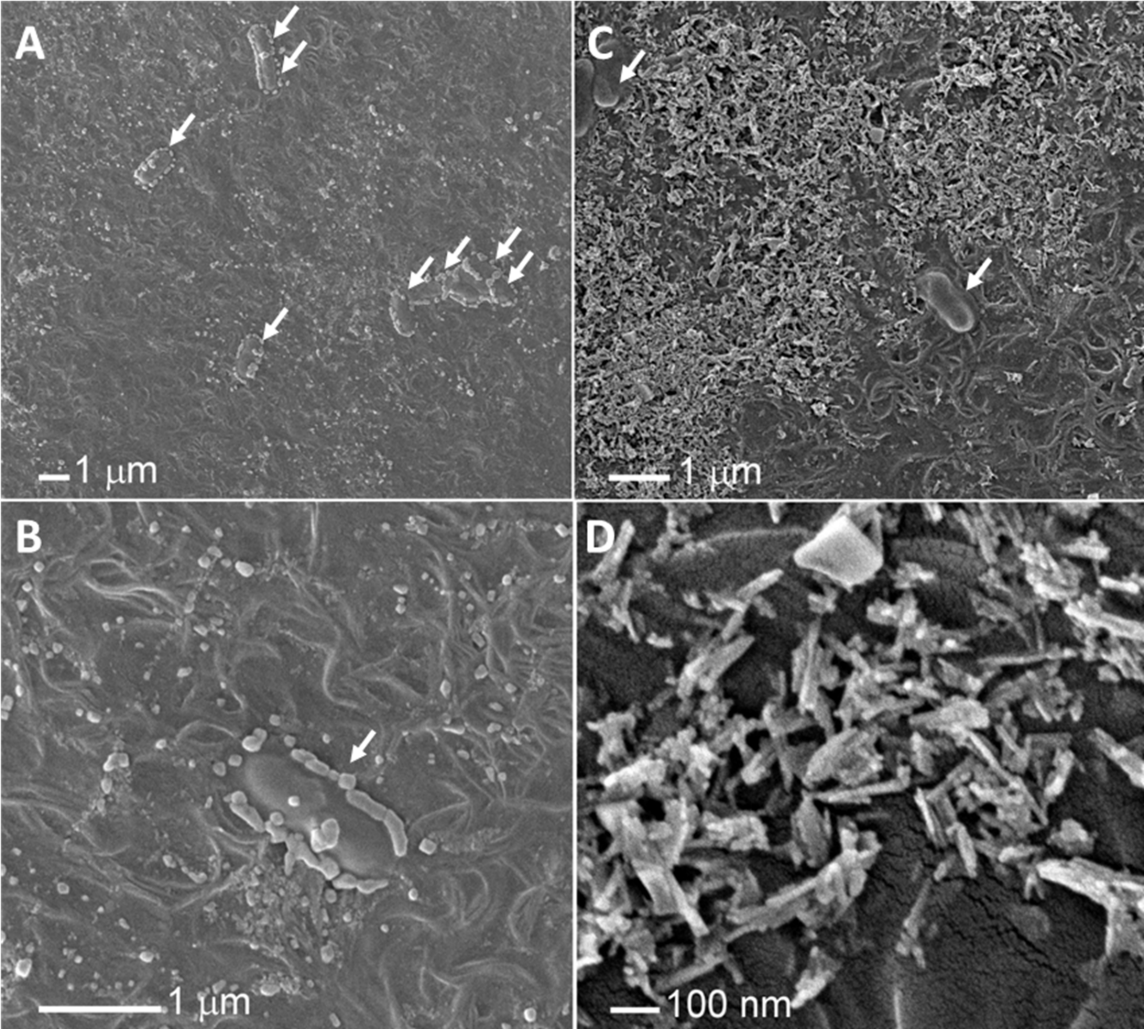
Scanning electron micrographs of Cu-TiO_2_NTs coated surfaces inoculated with *L*. *innocua*. A) uncoated surface; B) uncoated surface at higher magnification; C) Cu-TiO_2_NTs coated surface; D) Cu-TiO_2_NTs coated surface at higher magnification; bacteria are indicated by white arrows.

In addition to fluorescence microscopy, the Cu-TiO_2_NTs coated surfaces inoculated with *L*. *innocua* were inspected also at higher spatial resolution by scanning electron microscopy (SEM), which enables us to discriminate the nanotubes and bacteria based on the shape of objects. The micrographs confirmed that in agreement with fluorescence data shown above, individual bacteria were found mainly in those micro regions without or with much less of the nanomaterial coating. In contrast with the samples without the coating (Fig 6A and 6B), no clusters of organisms were observed on the Cu-TiO_2_NTs coated surfaces on any of the images, which further indicates the hostility of the coated surface to the microbes.

## Conclusion

The results presented here show that the TiO_2_ nanotube coated surfaces exhibit high photocatalytic activity when continuously illuminated with low intensity UVA light, efficiently disinfecting the surface against different and also pathogenic bacterial strains. Better understanding of the hydroxyl radical generation and its release from the surface as well as the incorporation of the copper atoms within the TIO_2_ matrix enabled us to minimize the rapid radical decay into hydrogen peroxide and transform the later back into hydroxyl radical. By this we were able to employ several-folds lower UVA light intensity to achieve the relevant inactivation of bacteria, allowing the application of inexpensive LED light sources. At the same time, maintaining the tube morphology helped us to achieve strong adhesion of the nanotubes to a preformed polymer surfaces, yielding a stable coating which can persist normal washing conditions in various media. Based on these results, we believe that promising new disinfection technology can be developed suitable for maintaining the surfaces clean in food storage and processing systems as well as in preventing persistent bacterial population in hospital infrastructure.

## Supporting information

S1 File Supplement to “Surface deposited one-dimensional copper-doped TiO_2_ nanomaterials for prevention of health care acquired infections”. (DOCX)
