# Supplementary material for: Surface deposited one-dimensional copper-doped TiO2 nanomaterials for prevention of health care acquired infections
Source: PLoS One. 2018 Jul 26;13(7):e0201490. doi: 10.1371/journal.pone.0201490 (PMC6062141; doi:10.1371/journal.pone.0201490)
Supplement: S1 File — (DOCX) [file pone.0201490.s001.docx]

**Supplement to:**

**Surface deposited one-dimensional copper-doped TiO_2_ nanomaterials for prevention of health care acquired infections**

Tilen Koklic^1,2^, Iztok Urbančič^3,1^, Irena Zdovc^4,2^, Majda Golob^4^, Polona Umek^1,2^, Zoran Arsov^1,2^, Goran Dražić^5^, Štefan Pintarič^6^, Martin Dobeic^6^, Janez Štrancar^1,2*^

^1^ Department of Condensed Matter Physics, Jožef Stefan Institute, Ljubljana, Slovenia

^2^ NAMASTE Center of Excellence, Ljubljana, Slovenia

^3^ University of Oxford, John Radcliffe Hospital, The weatherall Institute of Molecular Medicine, Human mmunology Unit, Headington, Oxford, UK

^4^ Institute of Microbiology and Parasitology, Veterinary faculty, University of Ljubljana, Ljubljana, Slovenia

^5^ Department of Materials Chemistry, National Institute of Chemistry, Ljubljana, Slovenia

^6^ Institute of Environmental and Animal Hygiene with Animal Behaviour, Veterinary faculty, University of Ljubljana, Ljubljana, Slovenia

^*^ Corresponding author

E-mail: janez.strancar@ijs.si (JŠ)


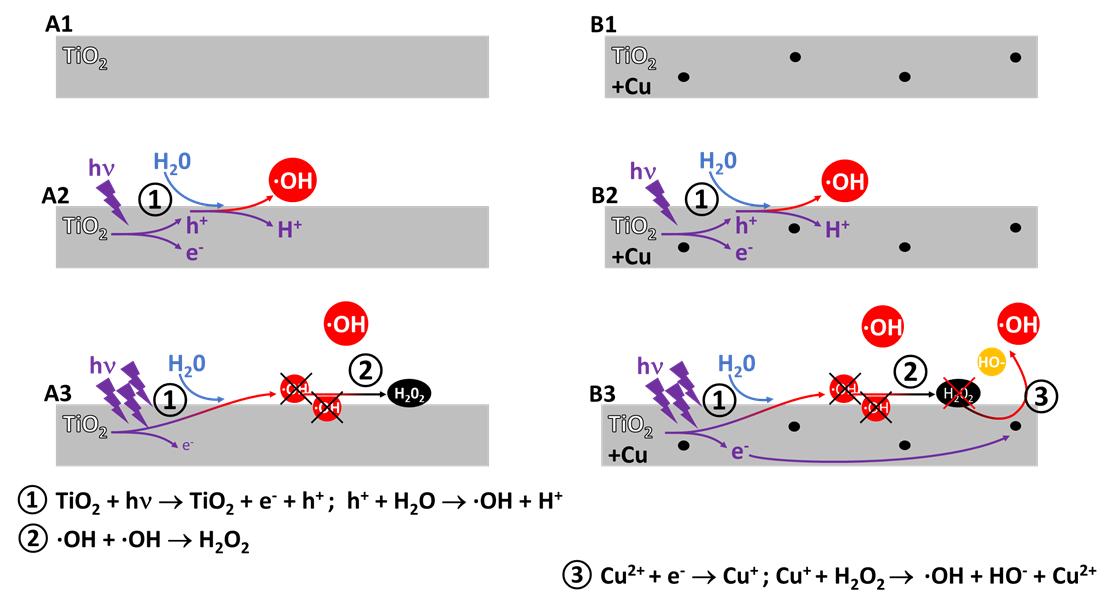


**Figure A. Literature review of reactions leading to production of OH radical on column A) TiO_2_; and column B) copper doped TiO_2_ surface**. A2 and B2) Low-intensity photoexcitation (middle row) leads to production of ٠OH radicals via photon absorption and water oxidation (reaction ①[1–4,4–8]); A3 and B3) High-intensity photoexcitation (bottom row) is accompanied by ٠OH radical annihilation and peroxide formation (reaction ②) [4], which can be turned back into ٠OH radicals by reduced-copper-mediated Fenton reaction (reaction ③[9,10], Cu-doped TiO_2_ case).

**Figure B. Identification and quantitization of hydroxyl radical production on Cu-TiO_2_NTs coated surafaces.** The activity of the anatase TiO_2_ nanotubes was measured in a low ionic strength buffer containing 30 % ethanol, using a spin trapping experiment with a 0.05 M spin trap DEPMPO.


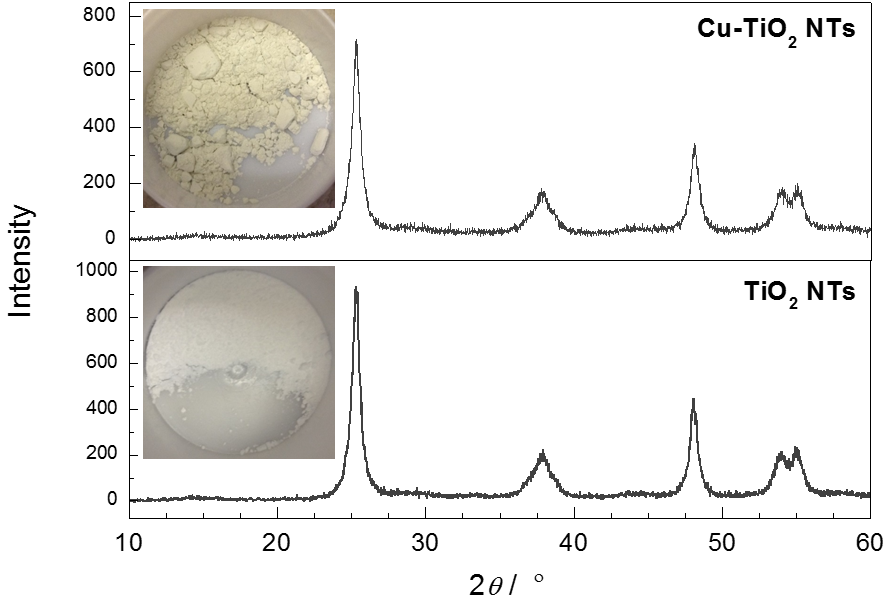


**Figure C. XRD patterns of copper doped TiO_2_ nanotubes (Cu-TiO_2_NTs, top) and pure TiO_2_ nanotubes (TiO_2_NTs, bottom).**


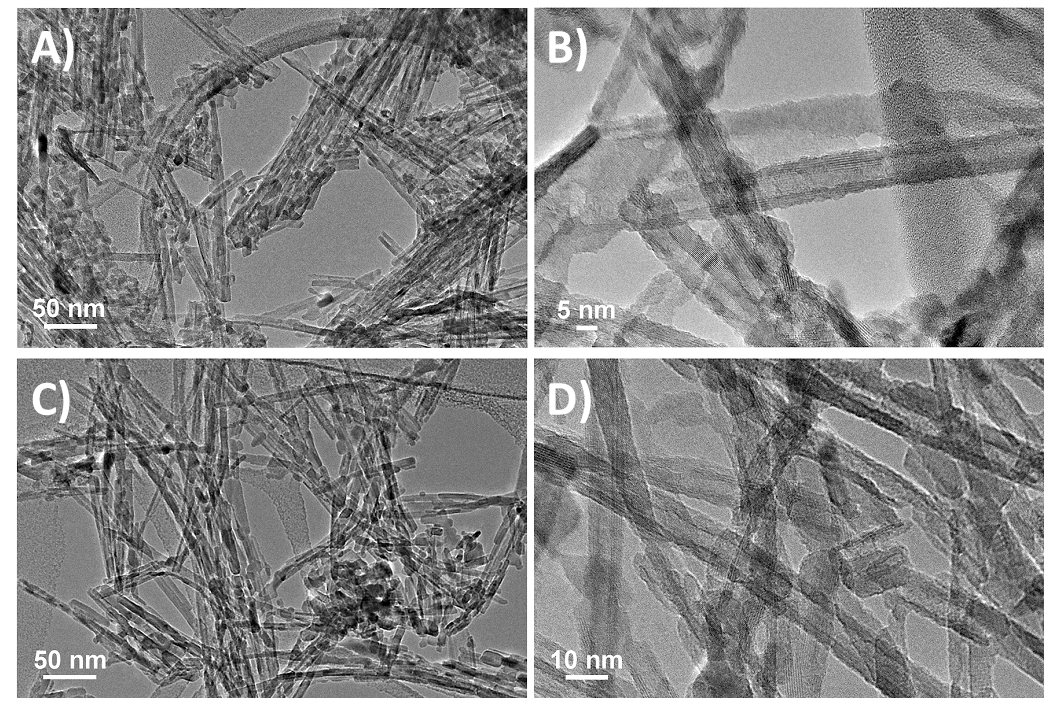


**Figure D. TEM images of Cu-TiO_2_NTs (A) and B)) and TiO_2_NTs (C) and D)).**


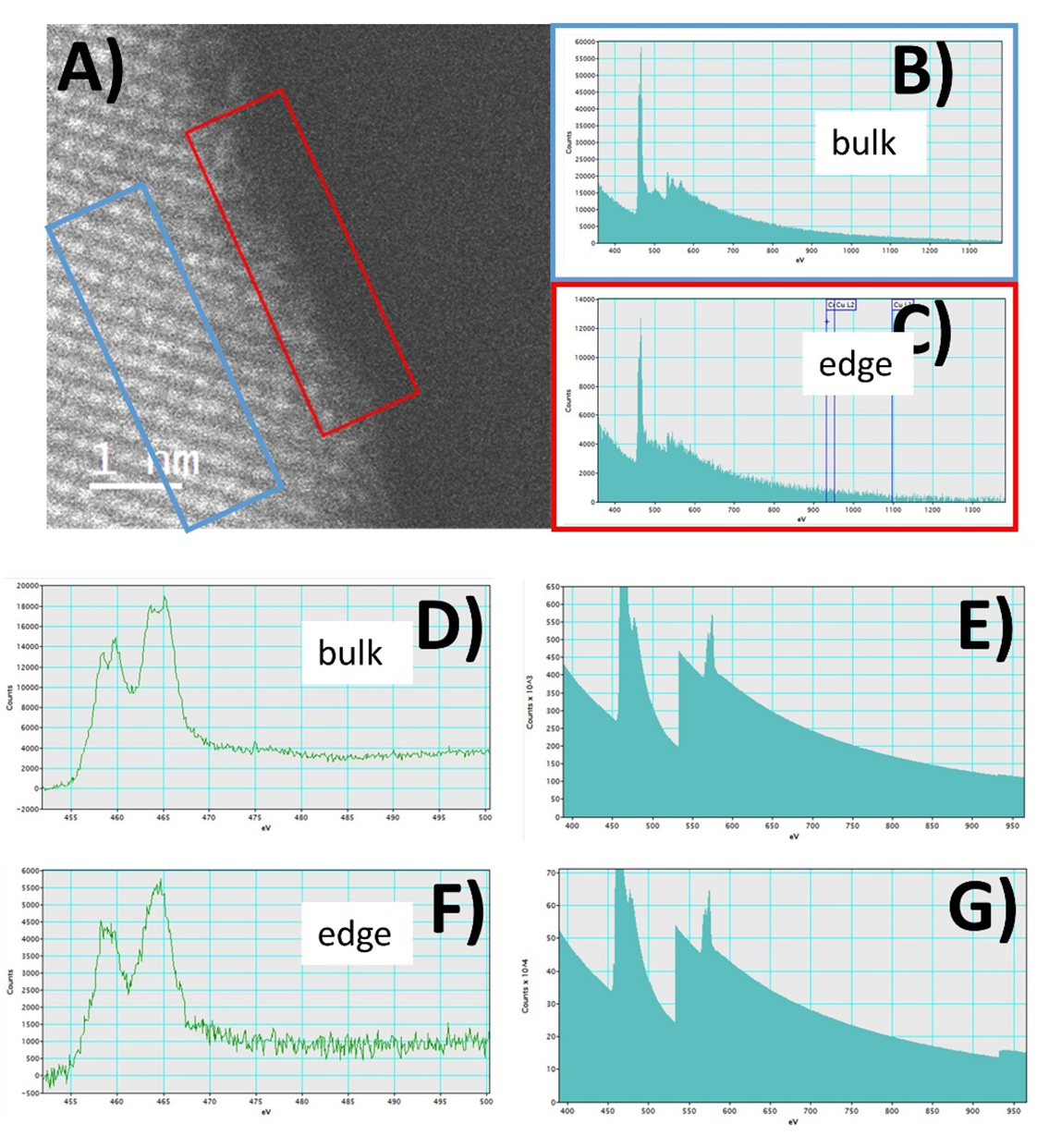


**Figure E. Transmission electron microscopy characterization of Cu-TiO_2_NTs**. (A) and (B) high resolution transmission electron microscopy (HRTEM); (C) high-angle annular dark-field imaging scanning transmission electron microscopy (HAADF-STEM); (D) annular bright-field scanning transmission electron microscopy (ABF-STEM); (E) HAADF-STEM at higher magnification; (F) ABF-STEM and; (E) HAADF-STEM of the CU doped TiO_2_ nanotubes.


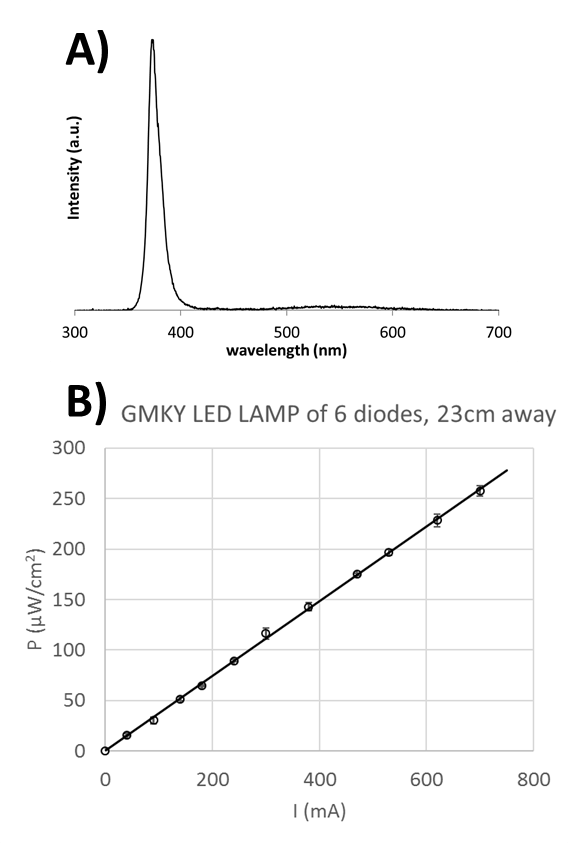


**Figure F. Emission spectrum and light intensity of the UVA LED lamp.** A) Emission spectrum of GMKY LED (U=3.4 V, I=280 mA, P_opt_=70mW, P_el_=0.95W) B) Intensity of LED lamp at the site of petri dish placement versus electrical current applied to the lamp. LED lamp consists of 6 GMKY diodes attached to 4 mm aluminum plate raised for 23 cm above illuminating surface.


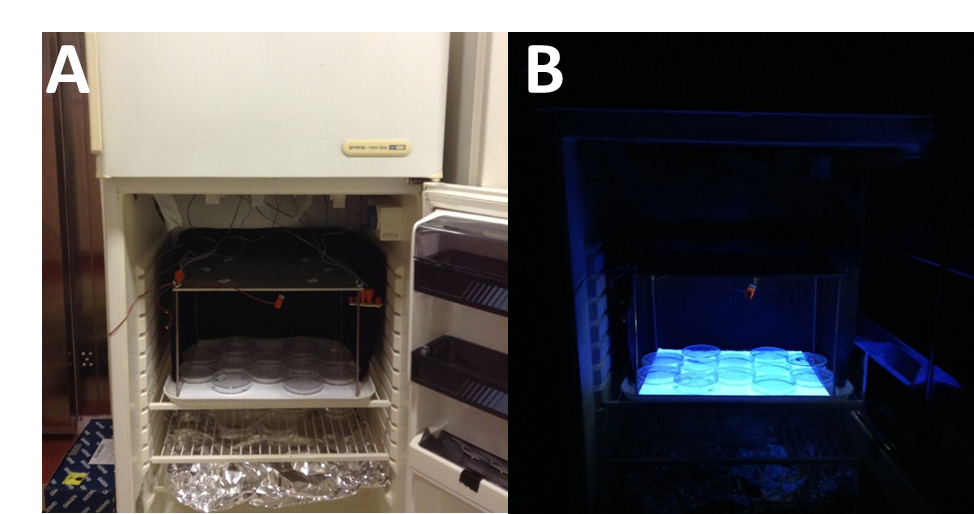


**Figure G. Experimental setup mimicking conditions in a refrigerator.** A) covered petri dishes in a refrigerator; (B) Illuminated petri dishes with LED diodes.


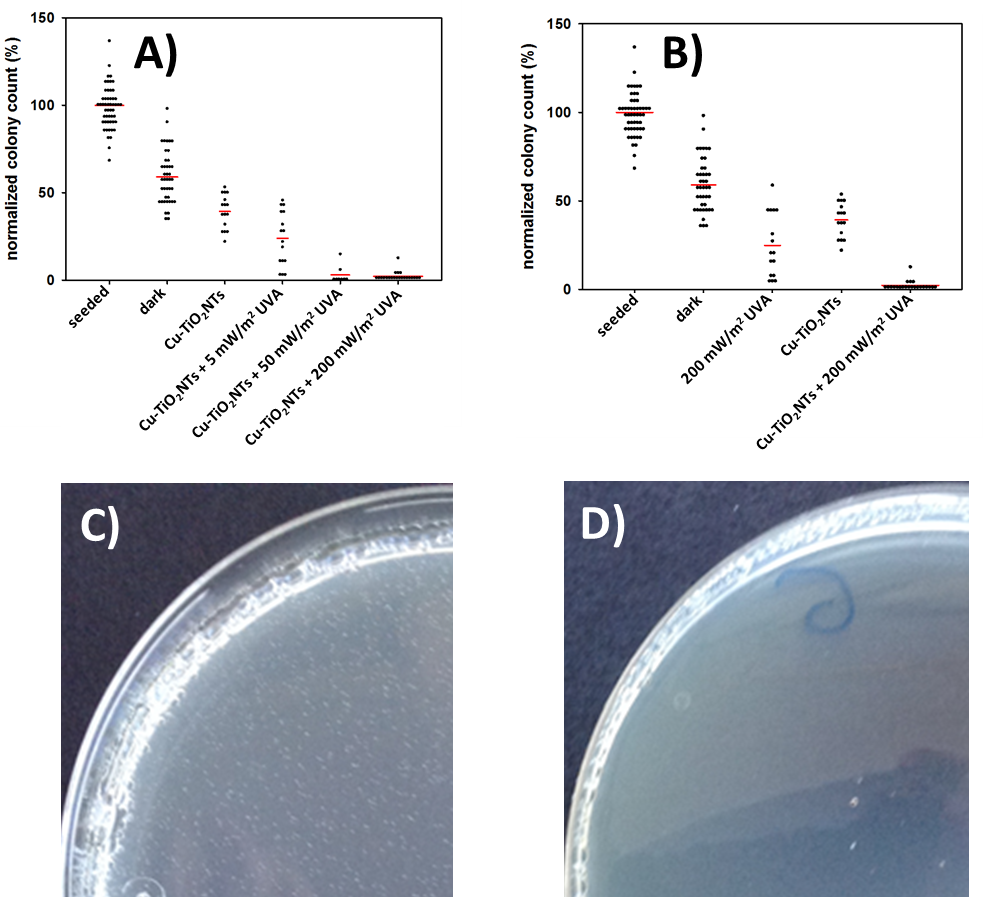


**Figure H. Survival of L. innocua on coper doped TiO_2_ nanotube coated petri dishes.** A) Normalized colony count of L. innocua versus intensity of UVA illumination; B) Normalized colony count of L. innocua at 200 mW/m^2^illumination versus all the corresponding controls. L. innocua was applied at density of around 100 microorganisms per petri dish (S= 50 cm^2^) covered with agar immediately after applying; after 24 hours in dark at 4˚C in an untreated petri dish (blank control); after 24 hours in dark at 4˚C in Cu-TiO_2_ coated petri dish (Cu-TiO_2_NTs); after 24 hours illuminated with 5 mW/m^2^ UVA at 4˚C in Cu-TiO_2_ coated petri dish (Cu-TiO_2_NTs + 5 mW/m^2^ UVA); after 24 hours illuminated with 50 mW/m^2^ UVA at 4˚C in Cu-TiO_2_ coated petri dish (Cu-TiO_2_NTs + 50 mW/m^2^ UVA); after 24 hours illuminated with 200 mW/m^2^ UVA at 4˚C in Cu-TiO_2_ coated petri dish (Cu-TiO_2_NTs + 200 mW/m^2^ UVA); after 24 hours illuminated with 200 mW/m^2^ UVA at 4˚C in untreated petri dish (200 mW/m^2^ UVA); C) L. innocua colonies (white colonies) on an untreated petri dish; and D) L. innocua colonies on Cu-TiO_2_ coated petri dish, formed after 3 day incubation at 37˚C.


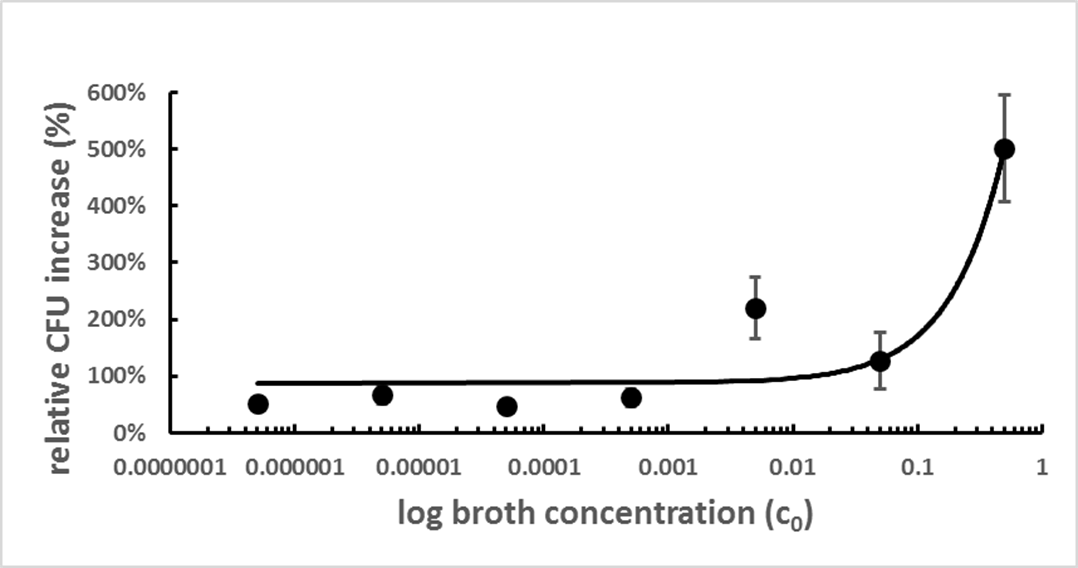


**Figure I. Effect of broth nutrients on L. innocua survival at 4˚C for 24 hours.** Even when L. innocua is diluted with saline about 50% of innoculated microorganisms survive 24 hours at 4˚C.


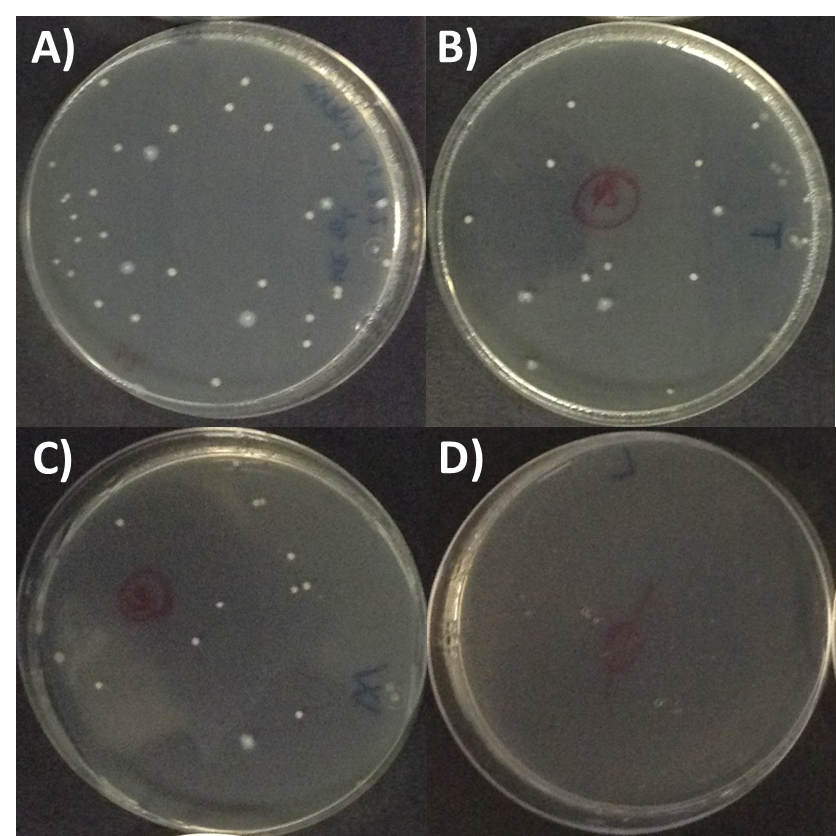


**Figure J. Representative images of colonies of L. innocua (white colonies) counted after 3 day incubation at 37˚C**. (A) an untreated petri dish and covered with agar immediately after inoculation (32 colonies); (B) an untreated petri dish and left at 4°C for 24 hours in dark (24 colonies, dark); (C) an untreated petri dish and illuminated with 0.3 W/m^2^ UVA light at 4°C for 24 hours (16 colonies, 200 mW/m^2^ UVA); (D) Cu-TiO_2_NTs coated petri dish and illuminated with 0.3 W/m^2^ UVA light at 4°C for 24 hours (0 colonies). On average 26±10 microorganisms were placed on each petri dish.


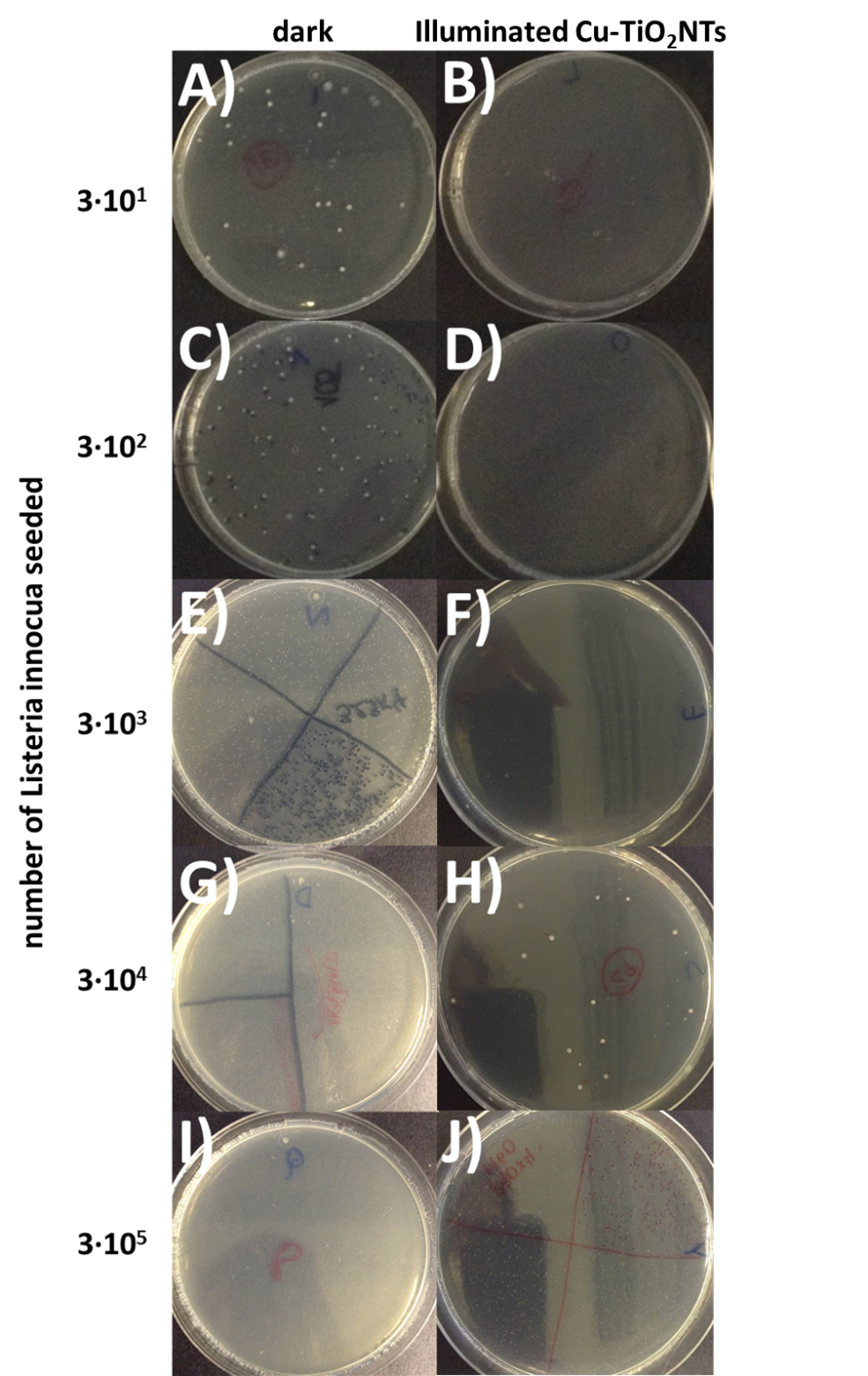


**Figure K. Representative images of colonies of L. innocua (white spots) counted after 3 day incubation at 37˚C at different bacterial densities.** Left column) on untreated petri dishes and left at 4°C for 24 hours in dark (dark); right column) Cu-TiO_2_NTs coated petri dish and illuminated with 0.3 W/m^2^ UVA light at 4°C for 24 hours (illuminated Cu-TiO_2_NTs).


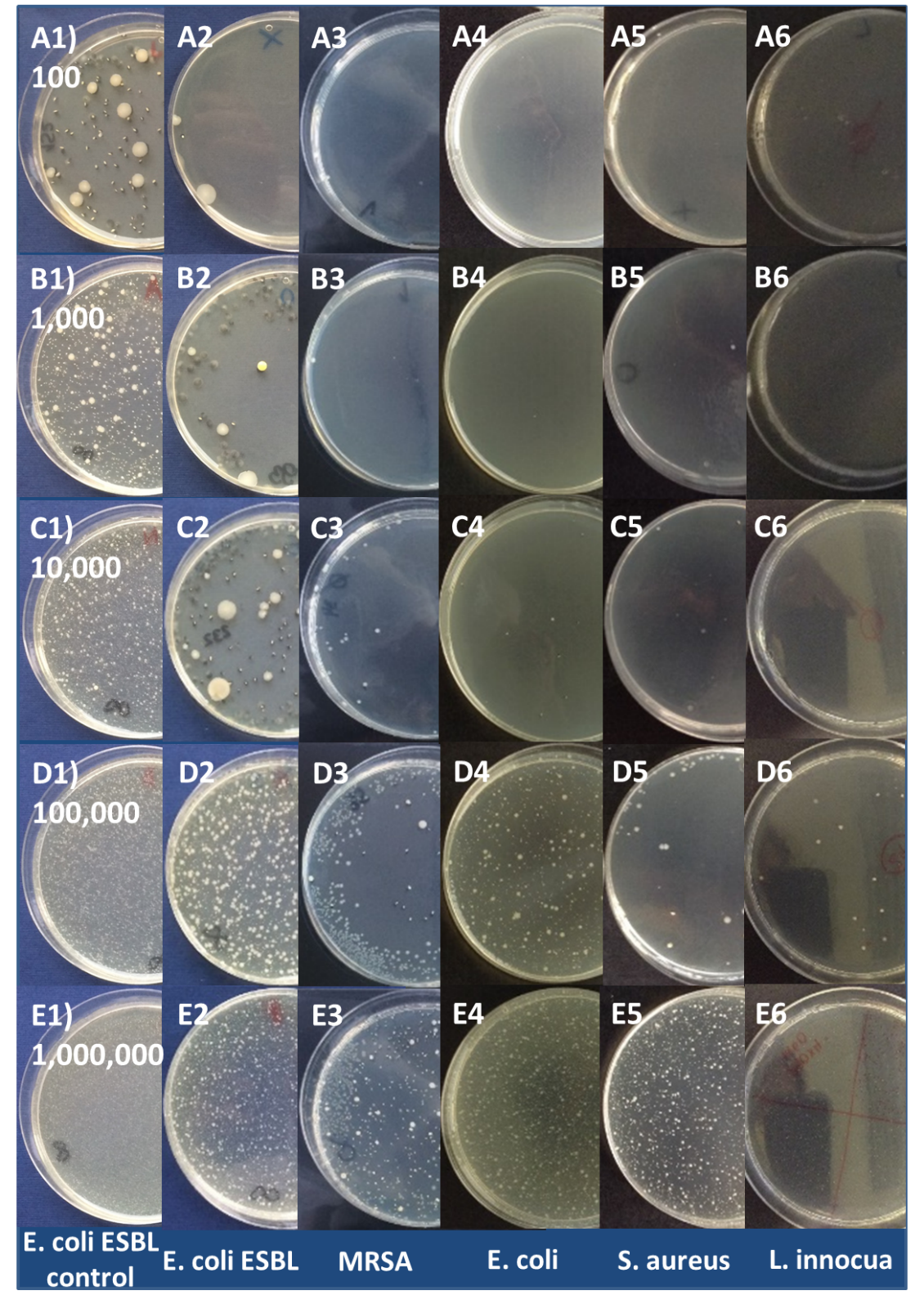


**Figure L. Representative images of colonies of different microorganism after 24 hour exposure to low intensity UVA light activated Cu-TiO_2_NTs coated surfaces at 4 ˚C.** Microorganisms were placed at different densities as shown in the first column.

**Figure M. Characterization of Cu-TiO_2_NTs coated surfaces inoculated with L. innocua using bleaching-corrected fluorescence microspectroscopy.** All samples were labeled with rhodamine B isothiocyanate (RITC). Fluorescence intensity (Intensity), wavelength of the fluorescence emission peak (Peak position), bleaching rate (Bleaching rate) images are shown together with the correlation graph of the peak position versus bleaching rate for A) uncoated surface, B) Cu-TiO_2_NTs coated surface, C) uncoated surface inoculated with L. innocua, and D) Cu-TiO_2_NTs coated surface inoculated with L. innocua. The spectrally contrasted images are color coded based on the local values of the associated fluorescence parameter (values for RITC spectral peak position (λ_MAX_) and bleaching rate (b), respectively). Lengths of the scale bars correspond to 10 µm on the sample. The white arrows mark individual bacteria that were identified by spectral analysis.

# References:

1. Hashimoto K, Irie H, Fujishima A. TiO2 Photocatalysis: A Historical Overview and Future Prospects. Jpn J Appl Phys. 2005;44: 8269–8285. doi:10.1143/JJAP.44.8269

2. Emeline AV, Ryabchuk VK, Serpone N. Dogmas and Misconceptions in Heterogeneous Photocatalysis. Some Enlightened Reflections. J Phys Chem B. 2005;109: 18515–18521. doi:10.1021/jp0523367

3. Herrmann J-M. Fundamentals and misconceptions in photocatalysis. J Photochem Photobiol Chem. 2010;216: 85–93. doi:10.1016/j.jphotochem.2010.05.015

4. Zhang J, Nosaka Y. Mechanism of the OH Radical Generation in Photocatalysis with TiO2 of Different Crystalline Types. J Phys Chem C. 2014;118: 10824–10832. doi:10.1021/jp501214m

5. Yi J, Bahrini C, Schoemaecker C, Fittschen C, Choi W. Photocatalytic Decomposition of H2O2 on Different TiO2 Surfaces Along with the Concurrent Generation of HO2 Radicals Monitored Using Cavity Ring Down Spectroscopy. J Phys Chem C. 2012;116: 10090–10097. doi:10.1021/jp301405e

6. Kakuma Y, Nosaka AY, Nosaka Y. Difference in TiO2 photocatalytic mechanism between rutile and anatase studied by the detection of active oxygen and surface species in water. Phys Chem Chem Phys. 2015;17: 18691–18698. doi:10.1039/C5CP02004B

7. Buchalska M, Kobielusz M, Matuszek A, Pacia M, Wojtyła S, Macyk W. On Oxygen Activation at Rutile- and Anatase-TiO2. ACS Catal. 2015;5: 7424–7431. doi:10.1021/acscatal.5b01562

8. Zhang J, Nosaka Y. Quantitative Detection of OH Radicals for Investigating the Reaction Mechanism of Various Visible-Light TiO2 Photocatalysts in Aqueous Suspension. J Phys Chem C. 2013;117: 1383–1391. doi:10.1021/jp3105166

9. Litter MI. Heterogeneous photocatalysis: Transition metal ions in photocatalytic systems. Appl Catal B Environ. 1999;23: 89–114. doi:10.1016/S0926-3373(99)00069-7

10. Cieśla P, Kocot P, Mytych P, Stasicka Z. Homogeneous photocatalysis by transition metal complexes in the environment. J Mol Catal Chem. 2004;224: 17–33. doi:10.1016/j.molcata.2004.08.043
